# Somatic therapies for treatment-resistant depression: ECT, TMS, VNS, DBS

**DOI:** 10.1186/2045-5380-2-14

**Published:** 2012-08-17

**Authors:** Cristina Cusin, Darin D Dougherty

**Affiliations:** 1 Division of Neurotherapeutics, Department of Psychiatry, Massachusetts General Hospital, 149 13th Street, Rm 2612, Charlestown, MA 02129, USA

**Keywords:** Treatment resistant depression, Electroconvulsive therapy, Transcranial magnetic stimulation, Vagal Nerve Stimulation, Deep brain stimulation

## Abstract

The field of non-pharmacological therapies for treatment resistant depression (TRD) is rapidly evolving and new somatic therapies are valuable options for patients who have failed numerous other treatments. A major challenge for clinicians (and patients alike) is how to integrate the results from published clinical trials in the clinical decision-making process.

We reviewed the literature for articles reporting results for clinical trials in particular efficacy data, contraindications and side effects of somatic therapies including electroconvulsive therapy (ECT), transcranial magnetic stimulation (TMS), vagal nerve stimulation (VNS) and deep brain stimulation (DBS). Each of these devices has an indication for patients with different level of treatment resistance, based on acuteness of illness, likelihood of response, costs and associated risks. ECT is widely available and its effects are relatively rapid in severe TRD, but its cognitive adverse effects may be cumbersome. TMS is safe and well tolerated, and it has been approved by FDA for adults who have failed to respond to one antidepressant, but its use in TRD is still controversial as it is not supported by rigorous double-blind randomized clinical trials. The options requiring surgical approach are VNS and DBS. VNS has been FDA-approved for TRD, however it is not indicated for management of acute illness. DBS for TRD is still an experimental area of investigation and double-blind clinical trials are underway.

## Introduction

Major depressive disorder (MDD) affects approximately 18 million people at any one time in the US alone, with a 17.1% lifetime incidence, and is associated with a high rate of morbidity and mortality [1].

Pharmacotherapy is effective in more than half of depressed patients; however, between 20% and 30% of patients suffering from depression may have “treatment-resistant depression” (TRD).

A recent review [2] compared five different models for classifying TRD, evolving from simply rating the adequacy of antidepressant trials to considering a more complex array of factors, including duration of illness, severity and treatment response. However, the validity and reliability of those models have been the focus of only a few studies, and their value for predicting outcome in clinical practice is still unclear.

The aim of this paper is to review new somatic therapies utilized in the treatment of TRD (defined broadly as failure to respond to two or more adequate trials of antidepressants in the current episode) in comparison with electroconvulsive therapy (ECT), considered the “gold standard” for patients with TRD.

## Methods

A literature search was performed using PubMed, Ovid Medline, Cochrane Database of Systematic Reviews and PsychINFO for articles published between 1990 and July 2011 with the following search terms: *ECT* or *electroconvulsive therapy, transcranial magnetic stimulation, TMS, depression, treatment-resistant depression, VNS* or *vagal nerve stimulation, DBS* or *deep brain stimulation, clinical trial*. Out of 807 articles retrieved, we included articles written in English that reported clinical trial results of an original sample, and 98 articles were ultimately reviewed in detail. The reference sections of the articles were checked for cross-references. For ECT, we included only the studies comparing efficacy of ECT with other devices. For DBS, given the absence of randomized controlled trials, we included all clinical trials and case reports.

## Results

### Electroconvulsive Therapy (ECT)

ECT consists of the application of an electric stimulus to the surface of the head, with the aim of inducing a seizure. The parameters of the stimulus can vary widely (pulsewidth from 0.3 to 1 msec, frequency from 20 to 120 Hz, duration of the stimulus 0.5-8 sec) and are adjusted for each individual patient, according to seizure threshold, clinical efficacy and side effects. The clinical efficacy of ECT in TRD is well established, with 60% to 90% rate of acute response in TRD [3], and it is indicated specifically in severe psychotic depression, catatonia and delirious mania.

ECT is associated with a number of common, but usually temporary, adverse effects such as arrhythmias, headaches, muscle aches, and nausea. Serious medical complications are exceedingly rare, even in patients with severe cardiovascular risk factors. The most common adverse effect of ECT is acute cognitive impairment lasting from few minutes to few days [4] or some form of amnesia, either anterograde or retrograde. More persistent deficits seem to be associated with older age, sine wave stimulation and bitemporal electrode placement [5]. The treatment is administered two to three times per week, and the patient requires an escort, due to driving restrictions after the treatment. ECT is available in academic and community settings, and the cost for each treatment session generally includes fees for the psychiatrist administering the treatment, the anesthesiologist, and the facility where the treatment is delivered. Although these costs vary considerably across centers, a course of 10 outpatient ECT treatments can cost approximately $10,000 to $15,000.

### Transcranial Magnetic Stimulation (TMS)

Transcranial magnetic stimulation (TMS) was introduced in 1985 as a technique to stimulate the cerebral cortex non-invasively [6]. A TMS device generates a strong magnetic field, inducing an electric current in a specific area, and this in turn induces intracerebral currents in associated neural circuits. The “single pulse TMS” has been utilized in research on localization of brain functions, while “repetitive TMS” (rTMS) has been used for treatment related studies. It is called “high frequency rTMS” if the stimulus frequency is above 1 Hz, or "low frequency rTM" if stimulus frequency is below 1 Hz. Low frequency rTMS is thought to inhibit cortical firing, while high frequency rTMS is thought to activate it.

The initial application of rTMS for depression was driven by functional imaging data showing reduced activity in the left prefrontal cortex (L DLPF) in patients with depression [7,8]. More recently, the investigators have focused on the idea of an imbalance in the activity of the frontal lobes (hypoactivity in the left frontal lobe and excessive inhibitory activity in the right frontal lobe), therefore leading to an alternative pattern for the treatment of depression combining low-frequency (suppressive) rTMS of the right dorso-lateral prefrontal cortex (R DLPF) with rTMS at high frequency to the L DLPF.

TMS is a non-invasive technique, therefore not requiring anesthesia or driving restrictions, and it is safely performed as an outpatient procedure. In general, TMS is well tolerated, with no evidence of cognitive impairment and with exceedingly rare medical complications. Rare seizures have been reported in the past, but the risk is substantially decreased with current treatment guidelines. The most common adverse effects are headaches and facial pain. A therapeutic TMS course of treatments can be performed by a trained technician, and a typical TMS session lasts between 30 and 60 minutes; sessions take place 5 times a week, usually for a period of 4 to 5 weeks, for a total of 20 to 30 sessions. One of the major obstacles is the time commitment for patients, which entails daily visits of one to two hours to the facility where TMS is provided. The average cost of each session is between $300 and $400, leading to a total cost for an average course of TMS between $6,000 and $12,000.

#### Efficacy of TMS

Several meta-analyses of rTMS clinical trials have been published in the past ten years, with mixed results. The majority of TMS trials targeted the L DLPC with high-frequency stimulation, while only a few targeted the R DLPC with either low-frequency stimulation [9-11] or both [12-16]. The earliest meta-analysis [17] yielded a positive result for rTMS compared to a sham control; however, it included only five controlled studies and excluded the only negative depression trial for rTMS that had been published at the time [18].

In 2002, Martin and colleagues published a Cochrane review of all the TMS studies available to that date [19]. The authors found that high-frequency (> 1 Hz) prefrontal DLPF rTMS and low-frequency (≤ 1 Hz) R DLPF rTMS were statistically superior to a sham comparison, but only at one time point (immediately after two weeks of treatment), and this difference was not sustained two weeks later. The overall difference between active and sham treatment was not large, though statistically significant, and the authors concluded that there was “no strong evidence to support the benefit of rTMS as an antidepressant treatment.” Only one meta-analysis, which included 6 trials, found a negative result for high-frequency prefrontal DLPF rTMS compared to sham [20].

The largest controlled study was sponsored by a TMS equipment manufacturer and involved a double-blind, multisite trial with 301 medication-free patients with MDD who had not benefited from prior treatments. Subjects were randomly assigned to either active (n = 155) or sham TMS (n = 146) conditions [21]. The study found that active TMS was significantly superior to sham TMS at weeks 4 and 6, and its results were the basis for the Food and Drug Administration’s (FDA) approval of TMS for patients who had not improved following one antidepressant trial.

The most recent meta-analysis [22] included 34 clinical trials comparing rTMS to sham treatment. This analysis showed a statistically significant difference; however, other papers critically analyzed the methods applied in the clinical trials [23-25] and suggested that though the results showed strong statistical evidence of efficacy, the clinical results were not sufficient to recommend the implementation of TMS in clinical practice because of the methodological limitations and heterogeneity of the studies.

In fact, while the application site of TMS was the L DLPF in more than 90% of the studies, the stimulation parameters were extremely variable and the level of severity of the patients enrolled was generally low, as only 3 out of 34 studies included patients who had failed 2 or more antidepressants in the current episode.

To address those criticisms, the NIH sponsored a large multicenter study in 2009 [26]. In this trial, the parameters of rTMS were standardized to maximize the likelihood of robust antidepressant effect (five times per week with TMS at 10 Hz, 120% of motor threshold, 3000 pulses/session, for 4-6 weeks) and key methodological limitations were addressed (e.g., adequacy of masking, validity of sham treatment, training of raters and reliability of outcome evaluation, magnetic resonance imaging adjustment for proper scalp placement). The results of the primary efficacy analysis revealed a significant difference in the proportion of remitters (14.1% on rTMS versus 5.1% sham, p = .02); however, the total number of responders (n = 19) and remitters (n = 18) was overall very low and the number needed to treat (NNT) was 10. It is notable that in this study, as in the previous ones, most of the remitters had low antidepressant treatment resistance at the time of study entry.

#### Efficacy of TMS IN TRD

A few studies have investigated the use of TMS in patients with TRD specifically, usually in combination with antidepressant drugs. Overall the results reported have been positive, but it is very difficult to draw any conclusion because of small sample sizes, differing inclusion criteria (i.e., inclusion of patients with bipolar depression), variable treatment schedule and high dropout rate. Three were small open-label studies [27-29], and one was double-blind, sham-controlled study of the combination of TMS and escitalopram [30]. In all the aforementioned studies, the level of treatment resistance was low, and only one trial randomized patients with different levels of resistance to unilateral TMS, bilateral or sham [31]. Based on published data, the role for TMS in the treatment of TRD is still unclear.

#### TMS comparison with ECT

One question that is particularly relevant for the clinician is how TMS compares to ECT in patients with TRD. There have been six prospective studies to date comparing TMS and ECT in patients with a major depressive episode (MDD or bipolar depression) [32-37]. The sample size in each study was usually small, between 25 and 42 patients, and out of those six, three studies indicated superiority for ECT and three found no difference between the two devices. The results were reviewed in a meta-analysis, including a total of 113 TMS patients and 102 ECT patients, yielding a combined 38.0% rate of response or remission rate for TMS versus 58.8% for ECT. This resulted in a highly significant difference in favor of ECT (chi sq = 9.267, *p* = 0.0023) [38]. Major criticisms common to all these studies were that they generally utilized low energy and low frequency for both ECT and TMS, included patients with psychotic features, and did not adequately describe blinding techniques. Despite these limitations, it seems that ECT has superior efficacy, in particular for those patients with severe depression, psychotic features and higher level of treatment-resistance.

#### TMS summary

In 2008, the U.S. FDA approved rTMS as a treatment for adults with MDD who “have not responded to a single antidepressant medication in the current episode.” Since then, one large NIMH-sponsored, multicenter, randomized, sham-controlled study has been conducted [26], and this trial showed a statistically significant difference between the treatments, but overall low rates for both response and remission.

In general, TMS is indicated in adults with MDD who “have not responded to a single antidepressant medication in the current episode.” However, numerous questions still persist about the magnitude of the efficacy of TMS in patients with TRD.

Due to concerns that the antidepressant effects of rTMS are not sufficiently robust to be considered "clinically meaningful," insurance companies have often been reluctant to reimburse for TMS treatment

### Vagal Nerve Stimulation (VNS)

Vagal Nerve Stimulation (VNS) was approved for treatment-refractory epilepsy in Europe in 1994 and in the US in 1997. Early clinical observations of improvement of mood symptoms in epilepsy patients led to a pilot study of VNS on mood in patients with primary diagnosis of epilepsy. The Cyberonics VNS device currently available on the market consists of a titanium-encased lithium battery, a lead wire system with electrodes, and an anchor tether to secure leads to the vagus nerve. The generator is implanted in the left chest wall and connected with a lead to the left vagus nerve. The device is implanted in a procedure usually lasting 1 to 2 hours, either under local or general anesthesia. Following the surgery, the device is activated telemetrically by a wand connected to a handheld computerized device. The adjustment of treatment parameters, including selection of stimulus intensity, duration and on/off interval, is non-invasive and is performed using an external telemetric wand. Stimulator settings are programmed to deliver intermittent stimulation with a current of 0.25–3.0 mA, a frequency of 20–50 Hz, a pulse width of 130-500 ms, and duty cycle (e.g. 30 seconds on, 5 minutes off). The mechanism of action of VNS is not completely understood and its effects are thought to be relatively slow, in the order of months; for this reason, VNS is not indicated for the relief of acute depressive exacerbations.

The safety of VNS is well established from its use in the treatment of epilepsy. The side effects of VNS are generally mild and are associated with stimulation (i.e., the “on” phase of the cycle). Because VNS causes stimulation of the superior and recurrent laryngeal nerves, it is frequently associated with problems ranging from alteration of the voice, coughing, throat pain and hoarseness (very common), reversible bradyarrhythmias and obstructive sleep apnea. Infections associated with the hardware are infrequent but possible. Regarding psychiatric adverse effects, the rate of stimulation-induced switch to mania or hypomania in the VNS trials was low in the pivotal trial by Rush and colleagues [39] (<0.01% at one year), and those symptoms subsided with adjustment of stimulation parameters.

#### VNS Efficacy IN TRD

In psychiatry, VNS has been developed primarily as adjunctive therapy for patients with TRD who have failed to respond or have declined ECT; therefore, no comparison with ECT is available. The first open-label trial of VNS in depression enrolled 30 patients with a major depressive episode (MDD or bipolar depression) who had failed at least two adequate antidepressant trials (the average number of failed trials was 4.8 ± 2.7). VNS was used as augmentation strategy, and the subjects received 10 weeks of stimulation in combination with their medication regimen. The results at 10 weeks were a response rate of 40% and a remission rate of 17% [40].

The second pilot study combined the initial study cohort with another sample of 30 patients, followed for 12 weeks, and obtained a response rate of 30% and remission of 15% [41]. The higher degree of treatment resistance in the second pilot study may have been a significant factor in the less favorable outcome. Similar results were reported by Schlaepfer and colleagues in an open-label European study including 74 patients [42]. The striking finding in the long-term follow-up of both those studies was the apparent increase over time in response and remission rates [43,44].

The largest (n =235) randomized, sham-controlled, multicenter study of adjunctive VNS, which enrolled patients with higher level of treatment-resistance (failed to respond to two or more monotherapies and two augmentations, failure to respond to ECT), did not find a significant difference between active and sham groups at 12 weeks (15% and 10%, for response and remission respectively) [39]. However, follow-up observations of this cohort suggested a cumulative beneficial effect of treatment over time, reaching response rates between 27% and 34% and remission of 15% at one year [45], with similar outcomes for patients with MDD and BP depression [46]. It is very interesting that in the long term follow-up, the authors reported a decline in suicide attempts, diminished levels of suicidal ideation, and fewer hospitalizations for depression in patients with VNS compared with patients with the same level of symptom severity but taking medications [47].

Overall the number needed to treat (NNT) for VNS ranged from 4 to 10, which, given the level of treatment resistance in this population, is clinically significant. In 2005, based on the clinical trial data, the FDA approved the use of VNS as an adjunctive therapy for treatment-resistant depression in adult patients who have failed 4 or more medications. Unfortunately the same data were deemed insufficient for approval of reimbursement under Medicare/Medicaid, therefore seriously limiting the access of patients to VNS.

The estimated cost for the surgical implantation of VNS in day surgery is on the order of $40,000 to $45.000. Furthermore, this estimate does not include the cost of post-operative device adjustments ($350-$620), which are rather frequent at the beginning of treatment and may significantly impact the total costs of the treatment.

#### VNS summary

The safety of VNS is well established from its use in the treatment of epilepsy for almost two decades, and it appears to be effective in patients with MDD or Bipolar II disorder with low to moderate, but not extreme, antidepressant resistance. VNS is usually considered as an adjunct to pharmacologic treatment, and it can safely be combined with ECT in case of an acute relapse. Because its effects take much longer to appear compared to antidepressants or ECT, VNS cannot be considered a treatment for acute TRD. The major barriers preventing its diffusion in clinical practice of this device are the elevated cost and the lack of reimbursement by insurance providers in the US at present.

### Deep Brain Stimulation (DBS)

DBS is a reversible neurosurgical procedure, consisting of implanting electrodes at specific anatomical locations and delivering an electrical impulse of variable intensity and frequency through those electrodes. DBS is thought to induce an electrical field that alters complex firing patterns of the neurons and thus modifies activity in the neuronal circuits. DBS has been utilized for treatment-refractory essential tremor and is approved for Parkinson’s disease [48] and dystonia. In 2009, DBS was approved for treatment of otherwise intractable obsessive-compulsive disorder (OCD) in Europe and in US.

Since the early 1960s, it has been observed that both acute and chronic stimulation could induce mood changes, including hypomania, dysphoria, and anhedonia. These observations led to the development of clinical trials to test the possible efficacy of DBS in treatment-refractory mood disorders. A number of research groups are currently investigating different sites for implantation of electrodes (for a complete review see [49]).

1. Subcingulate –Broadmann area 25 (SCG 25) - Mayberg and colleagues have demonstrated the integral role of the subgenual cingulate cortex in both normal and pathological shifts in mood [50]. Moreover, other studies have indicated an association between a clinical response to antidepressants and decrease in metabolism in limbic and striatal areas (subgenual cingulate cortex, hippocampus, insula, and pallidum) and increase in metabolism in dorsal cortical areas (prefrontal, parietal, anterior, and posterior cingulate cortex).

2. Ventral anterior internal capsule/ventral striatum (VC/VS) - The dorsal and ventral prefrontal cortex (PFC) have been found to be dysfunctional in neuroimaging studies of patients with MDD. These regions are nodes in a corticostriatalthalamocortical (CSTC) circuit that also includes components of the striatum and thalamus. The VC/VS target for DBS was developed following studies of gamma-knife capsulotomy for obsessive-compulsive disorder (OCD) and investigated in early studies by Nuttin [51] and Greenberg [52]. In those patients with primary OCD, a significant improvement was noticed in depressive symptoms, leading to the investigation of this target in MDD. Functional neuroimaging studies in DBS subjects showed activation of the ventral pre-frontal cortex, striatum, and thalamus during acute stimulation of the VC/VS target [53].

3. Nucleus accumbens (NAC) and ventral striatum - The reward circuitry of the ventral striatum and NAC has been associated with drug addiction and depression. The NAC and ventral striatum receive projections primarily from the anterior cingulate cortex, insular cortex, and orbitofrontal cortex. The NAC then projects to the dorsomedial nucleus of the thalamus by way of the ventral tegmental area, ventral pallidum, and substantia nigra—which in turn projects back to the prefrontal cortex, orbitofrontal cortex, anterior cingulate cortex, amygdala, and hypothalamus; this forms the limbic loop of the basal ganglia [54].

4. Inferior thalamic peduncle (ITP) - The ITP is a bundle of fibers that connects the thalamic system to the orbitofrontal cortex. This system is thought to induce electrocortical synchronization and to allow the inhibition of irrelevant stimuli, thus allowing selective attention. The ITP has been identified by Velasco and colleagues [55] as a potential stimulation target for TRD. At present there is only one case report of successful DBS electrode implantation in the ITP for refractory depression [56].

5. Lateral habenula (LH) - The LH has been proposed as a putative target for DBS. The habenular nuclear complex projects to the locus ceruleus, medial dorsal frontal cortex, orbitofrontal cortex, insula and mesolimbic areas, and ventral tegmentum/brainstem. At present there is one case report of DBS at the LH for MDD [57].

#### Implantation of DBS

The implantation of DBS electrodes and batteries is a complex neurosurgical procedure. Under stereotactic guidance, two electrodes are placed in deep structures of the brain, relative to a set of anatomical landmarks. Two programmable neurostimulators are implanted in the chest area under the clavicle and are connected to the corresponding electrode by extension wires under general anesthesia. Systematic outpatient adjustment of stimulation parameters (active contacts, amplitude, duration, frequency) and frequent follow-ups are necessary, especially during the first 6-12 months after implantation. The rates of surgical complications are quite variable and include intracranial hemorrhage, infections, and rarely stroke, lead erosion and lead migration.

From the psychiatric point of view, there is a risk of developing manic or hypomanic symptoms, anxiety, or worsening depression, but those symptoms are in general transient and respond to modification in parameters of stimulation. Suicides have been reported in patients with movement disorders and depression implanted with DBS at different targets.

DBS in treatment-resistant patients requires a dedicated multidisciplinary team of neurosurgeons, psychiatrists, neuropsychologists and support staff. The estimated cost of the implant is approximately $200,000 -$250,000, including device implantation and hospital stay but not including preoperative assessments, travel expenses, or costs possibly related to surgical complications. Replacements of the batteries add to the total burden for the patient, being necessary every 12-24 months on average, with an estimated cost of approximately $95,000 each time for hardware replacement and surgery. It is difficult to estimate the total additional cost of the sessions necessary to adjust the parameters, between $350 and $650 with an initial frequency of every two weeks, then every month on average for the first year. A more comprehensive evaluation of costs of DBS in patients with Parkinson’s disorder outlined the importance of using broader outcome measures of quality of life to calculate the true impact of DBS on patients and their caregivers [58].

#### DBS Efficacy IN TRD

To our knowledge, preliminary double-blind, randomized, sham‒controlled studies are being conducted for two targets, SC25 and VC/VS, and the results have not been published yet. In literature, open-label studies have been published for three targets: SC25, VC/VS and NAC.

1. SCG25

The early open-label study recruited 20 patients [59] with an MDE and a level of treatment-resistance of 4 or 5. For this cohort, the follow-up has reached a mean duration of 42 months [60]. The percentage of patients who responded was 62.5% after 1 year, 46.2% after 2 years, 75.0% after 3 years, and 64.3% at last follow-up visit. Remission rates over time also remained consistent with rates of 18.8% after year 1, 15.4% after year 2, 50% after year 3, and 42.9% at last follow-up visit. More recently, a Spanish group published the results of another independent open-label trial of DBS implanted at SCG25 in 8 patients with a level of TRD of 5 [61]. In this sample, the response and remission rates were respectively 87.5% and 37.5% at 6 months and 62.5% and 50% at one year.

2. VC/VS

In a sample of 26 patients with intractable OCD who received VC/VS DBS treatment, the majority also had comorbid depression. In this cohort, after 36 months of VC/VS DBS stimulation, average HAM-D scores decreased by 43.2%, and 14 of the 26 patients met criteria for remission. Based on previous experience with OCD patients[62], an open-label, multicentric trial of VC/VS DBS for severe TRD was conducted with 15 patients with level of treatment resistance of 5 and mean duration of illness of 21 ± 11 years [63]. The results were extremely positive, with response rates of 40% at 6 months and 53.3% at 24 months, while remission rates were respectively 20% and 40%. In general, the DBS treatment was well tolerated, and no patients withdrew from the study.

3. NAC

In 2008, Schlaepfer and colleagues described bilateral implantation in the shell and core of the NAC in 3 patients with TRD [64]. In 2010, the same group published the results for open-label 1-year study including 10 patients [65]. All of the patients enrolled in the study had a history of recurrent or chronic TRD and a high level of treatment‒resistance. The results were similar to those obtained with DBS at other targets, with 50% of the patients reaching the threshold for response at 12 months.

#### DBS summary

DBS for TRD is an experimental area of investigation. Given the elevated costs and the risks related to the surgical procedure, DBS has been limited to the most treatment-refractory cases of depression. The data on efficacy in TRD are limited to a series of open-label studies, and rigorous double-blind trials are being conducted to establish its role in the management of patients with moderate to severe illness. DBS is not a treatment indicated for acute worsening, as the effects of stimulation can take weeks to months to manifest. Moreover the interactions between stimulation and medications need to be carefully managed. In the case of relapse, DBS can be combined successfully with previously ineffective antidepressant treatments, including medications, ECT and psychotherapies.

## Review and conclusions

Treatment-resistant depression has been associated with poor clinical outcomes, impaired long- term social functioning, and high rates of medical comorbidity and mortality.

Device-based therapy can be successfully integrated into the algorithm for management of TRD, in conjunction with the continuation of antidepressant regimens. Each device has specific indications according to the level of the patient’s treatment resistance. A summary of the features of each device as compared to ECT is presented in Table 1.

**Table 1:** Comparison of devices for Treatment Resistant Depression (TRD). ECT : Electroconvulsive Therapy; TMS Transcranial Magnetic Stimulation; VNS : Vagal Nerve Stimulation, DBS; Deep Brain Stimulation

	**ECT**	**TMS**	**VNS**	**DBS**
**FDA status**	“Gold standard” in TRD	Approved for TRD after failure of one AD	Approved	Not approved – under investigation
**Reimbursed by insurance**	Yes	Few cases	No	No
**Requires surgery/multidisciplinary team**	General anesthesia	no	Yes	Yes
**Acute clinical efficacy**	yes	yes	No	no
**Approximate costs (USD)**	10-15,000 for 3-4 weeks	6-12,000 for 3-4 weeks	40-45,000 for surgery + device	200-250,000 for surgery + device
**Follow-up adjustments / maintenance (per visit, USD)**	200-800	200-400	350-620	350-620

TMS is safe and well tolerated, and it has been approved by the FDA for adults with depression who have failed to respond to one antidepressant. Given its favorable side effect profile, it may also be indicated for patients who are intolerant of medications. Its use in TRD is currently not firmly supported by clinical trials.

VNS and DBS have much higher costs and require a surgical approach; therefore they have been utilized primarily in patients with high, or extremely high, level of treatment-resistance, usually after the patient has failed a course of ECT. VNS has a well-established safety record deriving from its use in the treatment of epilepsy; it is FDA-approved for TRD and appears to be most effective in patients with MDD or bipolar disorder with low to moderate, but not extreme, antidepressant resistance.

DBS for TRD is an experimental area of investigation for which the optimal neuroanatomical targets and stimulation parameters have yet to be determined. Given the elevated costs and the risks related to surgical implantation, DBS has been utilized in the most treatment-refractory cases. Future directions for the clinical development of TMS involve designing double-blind randomized controlled trials of augmentation of antidepressant therapy in patients who are drug-naïve or have moderate depression severity and designing protocols for long-term maintenance. For VNS, papers about its long-term benefits and limited adverse effects are being published, but it is unclear whether more controlled trials in depression will be pursued. For DBS, a series of open-label studies on DBS for TRD have been published, and double-blind research trials are underway.

As the number of patients treated with new devices increases, the ideal candidates and target population for each device will likely be established.

## Competing interest

The author(s) declare that they have no competing interests.

## Authors’ contributions

Dr. Dougherty receives funding from Medtronic (research and consulting/honoraria), Northstar (research), and Cyberonics (research).

CC and DD participated to the review of the literature and drafting of the manuscript. All authors read and approved the final manuscript.
