# Correction: Somatic therapies for treatment-resistant depression: ECT, TMS, VNS, DBS

**DOI:** 10.1186/2045-5380-3-1

**Published:** 2013-01-01

**Authors:** Cristina Cusin, Darin D Dougherty

**Affiliations:** 1 Division of Neurotherapeutics, Department of Psychiatry, Massachusetts General Hospital, 149 13th Street, Rm 2612, Charlestown, MA, 02129, USA

## Correction

Since the initial publication of the review article [1], a number of readers have contacted us to clarify potential erroneous information in the article. These comments focused on our review of the use of transcranial magnetic stimulation (TMS) for treatment-resistant depression (TRD). In the initial article, we concluded that TMS is well-tolerated and has been approved by the FDA for adults who have failed to respond to one antidepressant, but that its use in TRD was not yet supported by rigorous double-blind randomized clinical trials. It should be noted, that for the article, we searched PubMed and based our review on the several meta-analyses we found during this search.

1. The NIH-sponsored large multicenter trial [2] was published in 2010, not 2009. Also, note that the primary outcome measure of this study was remission, not response (as was the case in the Neuronetics study [3]). Most important, we erroneously reported that most remitters had low antidepressant resistance. In fact, the relationship between remission and treatment resistance was not significant. Also, the study cohort had an average of 1.5 failed research-quality adequate treatment trials (by Antidepressant Treatment History Form [ATHF] criteria), which translates approximately to 3 to 6 clinical medication trials, in the current episode and an average of 3.3 failed research-quality adequate treatment trials (approximately 9 clinical attempts) during their lifetimes. In conclusion, the data from George et al. [2] support the efficacy of TMS for TRD.

2. We were also provided a link (http://www.effectivehealthcare.ahrq.gov/search-for-guides-reviews-and-reports/?pageaction=displayproduct&productid=787) from the Agency for Healthcare Research and Quality (a division of the US Department of Health and Human Services) website that was not identified during our PubMed search. This review of primary data found that TMS demonstrates greater efficacy than sham control for TRD and found no statistically significant differences in depression severity and response rate in published studies directly comparing TMS to ECT.

We appreciate these comments from the readers and concur that, contrary to what is stated in our initial article, that there is substantial evidence supporting the efficacy of TMS for TRD.
